# The role of APTX4870 peptide in reducing cellular inflammatory responses by inhibiting *Mycobacterium tuberculosis*-derived mycolic acid-induced cytotoxicity

**DOI:** 10.3389/fmicb.2022.993897

**Published:** 2022-10-24

**Authors:** Xue Lin, Wei Jia, Gangning Feng, Yajing Su, Yuting Kang, Chen Zhang, Wenmiao Liu, Zhidong Lu, Di Xue

**Affiliations:** ^1^Institute of Medical Sciences, General Hospital of Ningxia Medical University, Yinchuan, China; ^2^Ningxia Key Laboratory of Clinical and Pathogenic Microbiology, General Hospital of Ningxia Medical University, Yinchuan, China; ^3^Department of Orthopaedics, General Hospital of Ningxia Medical University, Yinchuan, China; ^4^School of Education, Ningxia University, Yinchuan, China

**Keywords:** *Mycobacterium*, mycolic acid, multifunctional peptide, Nlrx1, cellular injury

## Abstract

Tuberculosis is a serious zoonotic disease caused by *Mycobacterium tuberculosis* (*M.tb*) and the *M.tb* complex. Mycolic acid is an extracellular carbohydrate polymer produced, secreted, and accumulated outside the cells of various *Mycobacterium tuberculosis* strains. Mycolic acid produced by Mycobacterium plays an important role in infection. However, there have been few reports on drugs that inhibit mycolic acid-induced cytotoxicity. The purpose of this study was to investigate the role of the panned peptide in Mycobacterium-derived mycolic acid (*M.tb*-MA)-induced cell injury. The heptapeptide (APTX4870) was isolated from various phage libraries using phage display (Ph.D-7, Ph.D-12, and Ph.D-C7C). The efficacy of APTX4870 against mycolic acid was demonstrated by evaluating clinical samples and conducting *in vitro* and Vivo. APTX4870 inhibited apoptosis, increased autophagy to decrease inflammation, and reduced *M.tb*-MA-induced lung damage. These findings suggest that this heptapeptide, which selectively targets *M.tb*-MA, might be exploited as a potential novel *M.tb* therapeutic treatment.

## Introduction

Mycobacterium tuberculosis (*M.tb*) is an obligate intracellular bacterium. In humans, *M.tb* infection produces leukopenia, tissue necrosis, and organ failure (Parrish et al., 1998; Barry III, 2001). As the infection time increases, *M.tb* may even cause cell death (Barry III, 2001; Brosch et al., 2002; Fitzgerald et al., 2005). To develop treatment methods, it is crucial to investigate the processes underlying *M.tb* infection (Vergne et al., 2004; Queval et al., 2017; Cohen et al., 2018; Gagneux et al., 2018). *M.tb* could reduce the flow rate of bronchial mucus (Billig et al., 2017; Osada-Oka et al., 2019), which is conducive to its colonization in the respiratory tract (Chai et al., 2020; Phelan et al., 2020). *M.tb* also decreases and eventually inhibits respiratory cilia swinging, which causes epithelial cell injury (Gagneux et al., 2006; Qiang et al., 2019). *In vitro*, *M.tb* impacts the function of a range of cells; nevertheless, the precise mechanism of its infection is unclear. (Fitzgerald et al., 2005; Chai et al., 2020) During *M.tb* infection, a multitude of virulence agents is released. Although there is literature on the infection process of these virulence factors, the pathogenic mechanism requires additional investigation (Chai et al., 2020).

*M.tb*’s cell wall is made up of two layers: the inner layer is made up of peptidoglycan, while the outer layer is mostly made up of mycolic acid (Huang et al., 2020; Verma and Subbarao, 2021). The outer layer functions as a protein permeation barrier and can offer resistance to lytic enzymes (Chai et al., 2020). *M.tb* has a surface layer outside its cell wall (Forrellad et al., 2013; Verma and Subbarao, 2021). Mycolic acid has been identified as the main secretory factor on the surface (Grzegorzewicz et al., 2012). The surface layer usually interacts with host cells (Sakai et al., 2021). The complex interaction between acid, lipid, and polysaccharide manufacturing in mycobacteria and cell elongation and division has been discovered (Grzegorzewicz et al., 2012; Huang et al., 2020). Mycolic acid is an intermediate in *M.tb*’s metabolic pathway that influences its development significantly. Because mycolic acid is required for *M.tb* pathogenesis, virulence, and persistence, it is an appropriate target for tuberculosis medicines (TB; Verma and Subbarao, 2021).

In this study, we used a phage display to isolate an *M.tb*-binding heptapeptide (APTX4870) from a Ph.D.-C7C library. In addition, we investigated the effect of APTX4870 on cellular damage and inflammation produced by *M.tb*-MA *in vitro* and *in vivo*.

## Materials and methods

### Phage panning and binding detection

Mycolic acid from *M.tb* (M4537) and n-hexane were purchased from Sigma-Aldrich (Saint Louis, MO, United States). Ph.D.-7, Ph.D.-12, and Ph.D.-C7C phage display peptide library kits were purchased from New England Biolabs (Beijing, China). The phage display libraries were panned based on a previously developed protocol (Xue et al., 2012; Chan et al., 2013). After four rounds of panning, high-binding 96-well ELISA plates were coated with 1 μg/well of *M.tb*-derived mycolic acid (*M.tb*-MA) for 12 h at 4°C (Vander Beken et al., 2011). After coating with *M.tb*-MA diluted in n-hexane, the hexane was allowed to evaporate for 2 h at 37°C. After n-hexane evaporation, incubate overnight at 4°C. The library was screened using antigen-coated. The purified phages were added to the wells and incubated for 2 h at room temperature (24°C ± 3°C). Phage clones were isolated from the pan’s eluted phages and showed significant absorbances in ELISA (Xue et al., 2012; Chan et al., 2013). DNA was extracted and sequenced from 45 *M.tb*-MA-binding clones. MEGA X software was used to perform cluster analysis on the amino acid sequences of the clones. One peptide corresponding to the encoded sequence in 45 clones was synthesized through standard solid-phase peptide synthesis with C-and N-terminal cysteine protections. The peptides were homogeneously purified using reverse-phase high-pressure liquid chromatography and analyzed using matrix-assisted laser desorption/ionization-time of flight mass spectrometry. 1 μg/μL BSA was used as a negative control.

### Clinical samples

Twelve clinical lung tissue samples, comprising six normal lungs and six lungs from TB patients, were obtained by surgical resection at the General Hospital of Ningxia Medical University, and informed consent was obtained from all the participants. Each patient gave written informed consent, and the study was approved by the respective institutional review boards. The study methodology conformed to the standards set by the Declaration of Helsinki. The age of Healthy patients was 46 ± 5 years, male, and the age of TB patients was 51 ± 5 years, male. Diagnosis of active TB was based on the following: positive mycobacterial culture result from the site of disease; or caseating granuloma on biopsy and/or clinical/radiological features consistent with active TB and a good response to therapy. Sarcoidosis patients all had mediastinal disease with compatible histology and clinical/radiological features to support a diagnosis of sarcoidosis and negative mycobacterial cultures. All the TB patients were not on antibiotic treatment. Patients were excluded if they were immunosuppressed either through disease (such as HIV, diabetes, or autoimmune diseases), medication, or had significant co-morbidities affecting the pulmonary system. For the experiments, we separated the lung tissue into three sections. A portion was used for mutant and APTX4870 binding ELISA detection. The tissue is ground with 0.9% normal saline and centrifuged at 14000× *g* in the specified experimental procedure. The supernatant was coated on an ELISA plate for antibody sandwich detection. Custom antibodies were designed against APTX4870 (1:1000), Mutant1 (1:1000), Mutant2 (1:1000), and Mutant3 (1:1000) using Custom Polyclonal Antibody.APTX4870 and other mutants were treated overnight with a part of the tissue fractions, and tissue proteins were lysed for WB detection. The remaining tissue was embedded for IHC detection. To determine the binding capacity of custom antibodies, we treated THP-1 macrophages 12 h after PMA induction with *M.tb*-MA (1 μg/μL). Co-incubation was performed with mutants and APTX4870. After extraction of cellular proteins, different antibodies were used for detection to determine the binding capacity of the antibodies.

### Animal and cell culture

All animal experimental procedures were performed at Ningxia University. The study was approved by the Ethics Committee of Animals of Ningxia University (2018–019). BALB/c mice (4-week-old, female) were purchased from the Shanghai Laboratory Animal Center of the Chinese Academy of Science and maintained temperature-controlled environment. THP-1 cell line was obtained from the Cancer Cell Repository (Shanghai Cell Bank, Shanghai, China). THP-1 cells were maintained in RPMI 1640 medium supplemented with 10% fetal bovine serum and 1% penicillin–streptomycin (Thermo Fisher, Rockford, IL, United States).

### Mice model

Bacterial lung infection models were created by instilling *M.tb*-MA in mice tracheas. The procedure was as follows: initially, mice were sedated with an intraperitoneal injection of 10% chloral hydrate at a dosage of 3 ml/kg. The mice were totally sedated before being secured with rubber bands and tapes to a wooden board (60° slope). After removing the mouse’s tongue, position the transmitted light source at the mouse’s neck, turn on the light source, carefully clean the throat secretions with a small cotton ball with tweezers, and adjust the animal’s inclination angle until it can be clearly seen through the mouth. Glottis separation is suitable. Insert the indwelling needle (consisting of an inner needle core and an outer tube) into the airway, then withdraw the needle core. The intubation is successful if the *M.tb*-MA put into the tube prior varies with breathing. Then, 50 μl of *M.tb*-MA(1 μg/μL) was gently instilled into the trachea using a 1 ml syringe linked to the indwelling needle tube, and the mouse was moved to ensure that the liquid was uniformly distributed in the animal’s left and right lungs. After 1 week of therapy, one mouse was dissected to detect the establishment of lung tissue damage. The APTX4870 (1 μg/μL) treatment group received the identical airway perfusion approach as the control group. PBS was utilized to perfuse the control group. Body weights of mice were measured on days 0, 3, 6, 9, 12, and 15. Food consumption in mice was measured on days 3, 6, 9, 12, and 15. Following the placement of 50 g of feed on the 0th day, the food intake of the mice was estimated on the 3rd day by weighing the decreased grams of feed, and so on. The procedure for measuring mice’s water consumption is the same as for eating. Mice were sacrificed with sodium pentobarbital and tissues were collected 15 days after treatment. For the experiments, we separated the lung tissue into four sections. 0.1 g tissue for WB, 0.1 g for tissue RNA extraction, and the remainder for HE and IHC.

### Western blot (WB) analysis

Mice lung tissues and cells were lysed using lysis buffer to obtain total cellular extracts. T-PER™ Tissue Protein Extraction Reagent was utilized to extract mice lung tissues (Thermo Fisher Scientific, United States). For lysis, use 5 ml of lysis buffer per 0.5 g of each lung tissue. Homogenize the tissue sample using the appropriate amount of T-PER Reagent. To pellet tissue debris, centrifuge the sample at 10,000× *g* for 5 min. Continue with downstream analysis after collecting the supernatant. We utilized M-PER® Mammalian Protein Extraction Reagent for cell cultures (Thermo Fisher Scientific, United States). 1*10^5^–1*10^6^ cells were lysed in 200 μl of lysis buffer. Pellet the cell suspension by centrifuging it at 2500× *g* for 10 min. Remove the supernatant. M-PER Reagent should be added to the cell pellet. To resuspend the pellet, pipette the liquid up and down. For 10 min, gently shake the mixture. Centrifuge the cell debris for 15 min at 14,000× *g*. Transfer the supernatant to a fresh tube for further examination. Total protein contents in cellular extracts were determined using a Pierce BCA Protein Assay Kit (Thermo Fisher, Rockford, IL, United States). The protein samples (20 μg) were separated using 10% sodium dodecyl sulfate-polyacrylamide gel electrophoresis (SDS-PAGE). HRP-conjugated secondary antibody or FLA9000 (Fuji Film, Minato, Japan) was used to visualize protein bands by enhanced chemiluminescence using ChemiDoc-It (UVP, Upland, CA, United States). Densitometric analysis was performed using ImageJ.^1^ Antibodies against IL-1β (1:1000), ULK1 (1:1000), mTOR (1:1000), LC3 (1:1000), and Beclin-1 (1:1000) were obtained from Wuhan Sanying, China. Custom antibodies were designed against APTX4870 (1:1000) and MA (1:1000) using Custom Polyclonal Antibody Production (Invitrogen, Rockford, IL, United States). Antibodies against cytochrome c (1:1000) were purchased from Abcam (Cambridge, United Kingdom).

### Histology and immunohistochemistry (IHC)

Optimal cutting temperature-embedded mouse lung sections were stained with hematoxylin and eosin (HE) as previously described (Hou et al., 2021). HE staining showed normal pulmonary tissues in mice in the control group. Sections were stained with hematoxylin and eosin (HE), to examine eosinophil infiltration Inflammatory index calculated and evaluated score using five-point scoring system. The severity of peribronchial inflammation was graded semi-quantitatively for the following features: 0, normal; 1, few cells; 2, a ring of inflammatory cells 1 cell layer deep; 3, a ring of inflammatory cells 2–4 cells deep; and 4, a ring of inflammatory cells of >4 cells deep. The score was considered by the inflammatory cell infiltration on perivascular regions and peribronchial of lungs. All lung tissue HE-stained scans of each group of mice were scored and quantified. All lung tissue HE-stained scans of each group of mice were scored and quantified. IHC staining was performed using antibodies as previously described (Rosenblatt et al., 2021). Custom antibodies were designed against Mutant1-3 using Custom Polyclonal Antibody Production (Abcam, Cambridge, United Kingdom). At least five slices per sample were prepared. Images were captured at 200× or 400× magnification using a Zeiss microscope and ZEN software.

### RNA isolation and quantitative real-time PCR (RTq-PCR)

Total RNA from mouse bronchial epithelial cells was isolated using TRIzol® reagent and reverse-transcribed using a Prime Script RT kit. PCR amplification was performed on an ABI 7500 Fast Thermal Cycler (Applied Biosystems, Waltham, MA, United States) using an SYBR Green PCR Kit (Takara Biotechnology Co. Ltd., Dalian, China). All primers used here are shown in Table 1. The RT qPCR technique was set to: 95°C (180 s), 95°C (10 s), 55°C (30 s), and 70°C (30 s), totaling 40 cycles. β-actin was utilized as an internal reference. The data were analyzed by Double Delta CT.

**Table 1:** qPCR primer sequences list.

Gene name	Forwards(5′-3′)	Reverse(3′-5′)
BAD	gtt ggc cct cac ctc ac	ctt ggc agc tct ctc acc
Caspase-3	tgg ccc tgt acg act tcc	cct gat ggt gtc ctc gct
IL-1β	ccc aga aga agc tga acg	ggg agc act cat gtc aaa a
IL-6	acc agc acc cca gca aa	tca cca gca gga aga agg c
TNF-α	gct ttt cag gca tcc tca t	cat cca gcc ttc cat tct t
Cyt-c	tct cca aca ccc gta cat c	cct cca agt cca taa ctt cct
IL-18	cca ggg cat tgt ctc cta	ggg gct tag ttt gct tcc
ULK1	gcg gat tga cat ttc tgt g	cat aag gca acg atc cca
PPAR-γ	cct ggg ttc cct ttc ctt	tcc tgg tcc ttg cat ctt t
IL-1α	ata gag cgt gaa ggc gaa	gca aag ttg gtg gtc ctg
IL-8	ata gcc ctc acc att tgc	ggg gac ttt cct tct tct g
NF-κB	gca gca acg aca cag aaa	agc ggt ggg taa tgg ag
LC3	caa ctg ctt cgt aat tgg c	tca tac gct tct ttc ttt cca
Bcl-2	cga gct gca cca tca tcc	aag gca ttg ggg ttg gc
Bxl-xl	ccc tcc ttc aga cac cct	ggt tgc cag cac ttc act
P62	cag tga tgc tgt gct atg aat	cag atg cct aag ttc ttc cac
IL-10	ctg agc tgg tct gaa ggc	gta cag gca ggc agg aag
IL-4	cag gtt ctg ggg aca gg	agg att cgc tct tgc gt
Atg5	tga tga ccc aca ctt cca	gac cca ttg ctt ctc agc
RAS	gca ccg agt tga ccg ta	gca gcg agt gtc ctt ctc
IL-12	caa taa cca ccc ctg acc	gcg cag aat gag atg agt t
MCL-1	ggt cct tgg tag agg gct a	ggt tag ggc agg gta gag a
IL-13	ggg agt gca ttg ggt tt	gtg ttt gcg aag cat cag
Bim	ggt tac atg gca gca aag a	agc gca gtt tca taa agc a
Beclin-1	aga ccc aaa cgc agg aa	aag ggc tga ccg aac aa
Atg13	agt acc ttg ttc caa tcc ca	gtt cct ctt tag cac cct ttc

### Cell viability assay

THP-1 cells were seeded in 96-well plates at a density of 5 × 10^3^ cells/well. Add PMA and make the final concentration of PMA as 100 ng/ml, the cells were seeded into a 96-well plate. After 12 h, the macrophages were successfully constructed under a microscope. Then, cell viability was detected using MTT assay according to the manufacturer’s instructions (Sigma-Aldrich, Saint Louis, MO, United States). The plate was read at 570 nm using a microplate reader (Model 680; Bio-Rad Laboratories Inc., Hercules, CA, United States). Untreated cells were used as controls, and the wells with phosphate-buffered saline (PBS) served as blank controls. The average optical density (OD) was subtracted from the OD of the sample and compared with that of control cells. We used the following formula to calculate the percent cell viability:

Percent cell viability = (average density (OD) of the treated group − blank/mean OD of control cells) × 100.

### Cell culture and treatment

THP-1 cells in logarithmic growth phase should be centrifuged and resuspended in RPMI-1640 culture media. Adjust the THP-1 cell density to 5 * 10^5^/mL after adding PMA and counting the cells under a microscope. After adjusting the final dose of PMA to 100 ng/ml, the cells were seeded onto a six-well plate and 2 ml of cells were added to each well; the macrophages were effectively created under a microscope after 12 h. The detection was carried out after the cells had been subjected to various treatments: PBS control, APTX4870 treatment, M.tb-MA (0.5 μg/μL) treatment, and M.tb-MA (0.5 μg/μL) + APTX4870 (1 μg/μL) treatment. The two components were combined 12 h before the cells were treated in the M.tb-MA + APTX4870 group.

### Scanning and transmission electron microscopy

5*10^6^ THP-1 cells were induced to differentiate into macrophages as described above. Then, cells were centrifuged, collected, and washed three times with PBS before discarding the supernatant. The cells were then fixed by incubating them in 4% glutaraldehyde overnight at 4°C. Cells were then treated with graded ethanol solutions (50, 75, 90, and 100%), air-dried, and coated with gold. 5*10^6^ THP-1 cells were induced to differentiate into macrophages as described above. The cell morphology was assessed using a scanning electron microscope (SEM; Inspect, FEI, Oregon, United States). Cells treated with 4% glutaraldehyde were post-fixed in 1% OsO4 and observed using a transmission electron microscope (TEM; JEM-1230, JEOL, Tokyo, Japan).

### Immunofluorescence

5*10^6^ THP-1 cells were induced to differentiate into macrophages as described above. The primary antibodies used were LC3 (1:50, Thermo Fisher, Rockford, IL, United States), Nlrx1 (1:100, Abcam, Abcam, Cambridge, United Kingdom), TUFM (1:100, Abcam, Abcam, Cambridge, United Kingdom), and IKKα (1:100, Abcam, Abcam, Cambridge, United Kingdom). DAPI (1:1000, Thermo Fisher, Shanghai, China) was used for nuclear staining. Immunofluorescence was performed as previously described (Hou et al., 2021).

### Measurement of intracellular reactive oxygen species (ROS)

ROS levels were assessed using a dihydroethidium staining kit (DHE, Thermo Fisher, Rockford, IL, United States) according to the manufacturer’s instructions. 5*10^6^ THP-1 cells were induced to differentiate into macrophages as described above. Then, cells were washed with PBS and stained with10 μM DCFH-DA for 30 min at 37°C under dark conditions. The level of ROS was determined by FACSCCalibur flow cytometer (BD Biosciences, United States).

### Pull-down assay

The Nlrx1-overexpressing plasmid (pcDNA3.1-Nlrx1) was constructed according to the ProFound Pull Down Polyhis Protein Protein Interaction Kit (Pierce/Thermo; Rockford, IL, United States) instructions. First, full-length Nlrx1 cDNA was cloned into pcDNA3.1. Lipofectamine 2000 reagent (Thermo Fisher, Rockford, IL, United States) was used to transiently transfect the clone into cells, following the manufacturer’s instructions. Pull-down assays were performed using the ProFound Pull Down Polyhis Protein Interaction Kit (Pierce/Thermo; Rockford, IL, United States).

### Statistical analysis

All data were obtained from at least three independent experiments under each condition. Excel and HiPlot^2^ were used for data analysis and graphic images. The *t*-test was used to compare the differences between the two groups (*p < 0.05, **p < 0.01, and ***p < 0.001).

## Results

### Generation and identification of *M.tb*-MA-specific binding peptide

**Figure 1:**
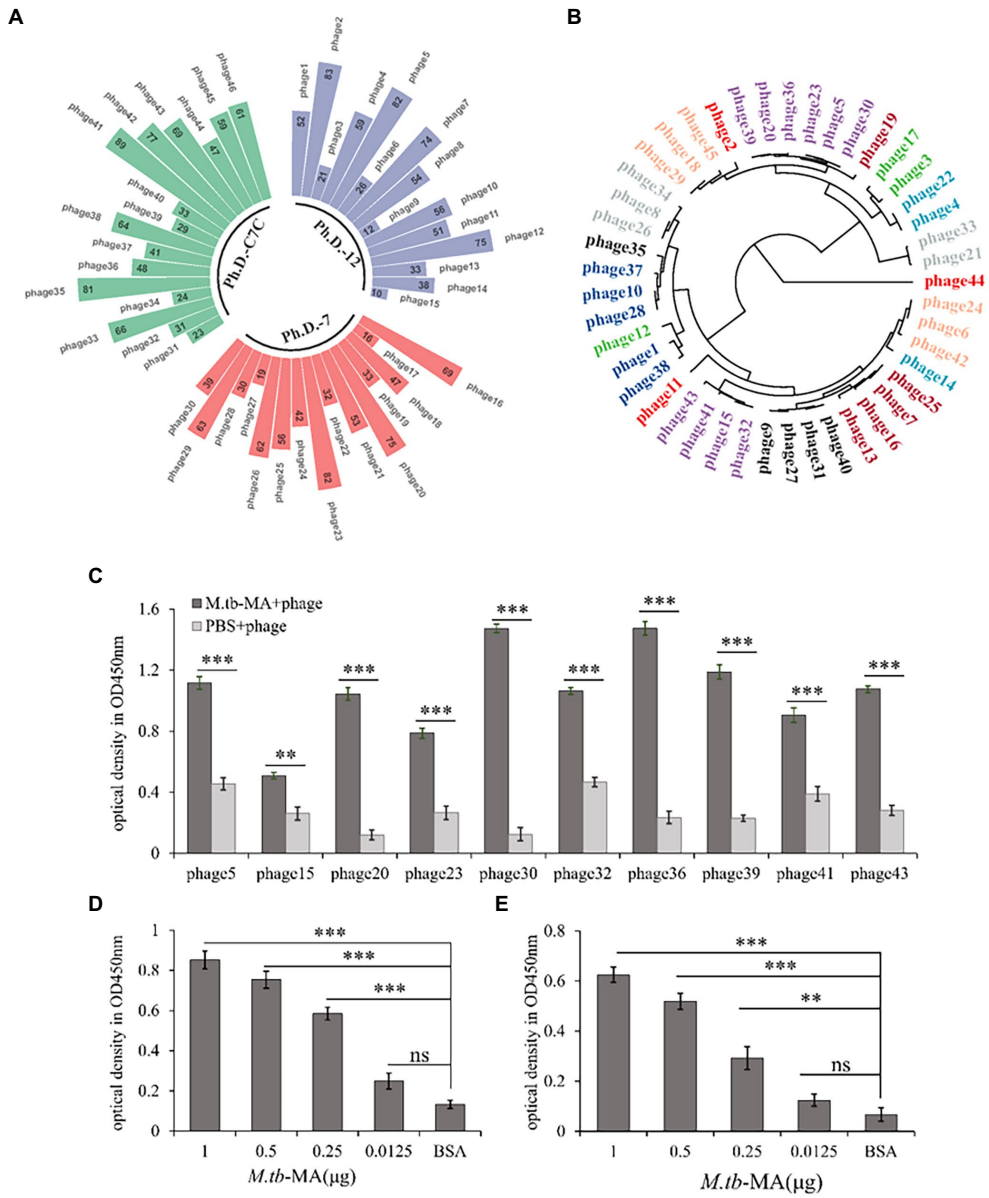
Generation and identification of *M.tb*-MA specific binding peptide. **(A)** *M.tb*-MA binding phage was obtained by screening different phage-displayed library shown in Nightingale rose chart. **(B)** Phylogenetic analysis of amino acid sequences of positive and higher binding phage. The tree was constructed using the neighbor-joining method; bootstrap of 1,000 replication (MEGAX). **(C)** 15 positive and higher binding phage clones panned by *M.tb*-MA were identified by ELISA (*n* = 3). **(D)** The binding of phages expressing APTX4870 peptide to *M.tb*-MA was tested *via* phage ELISA. Phages expressing APTX4870 were added to ELISA plates coated with BSA (negative control) and *M.tb*-MA (1, 0.5, 0.25, and 0.125 μg/well). Bound phages were detected using HRP-conjugated 9E10, and visualized with 3,3′,5,5′-tetramethylbenzidine (TMB). Absorbance was measured at 450 nm (*n* = 3). **(E)** His tag-conjugated APTX4870 was added to ELISA plates coated with *M.tb*-MA (1, 0.5, 0.25, and 0.125 μg/well). Bound APTX4870 was detected using HRP-conjugated anti-His tag antibody and visualized with TMB (*n* = 3). **0.01 < *p* < 0.05; ***p < 0.001.

The *M.tb*-MA binding phage was selected by screening 3 phage display libraries. ELISA was used to identify positive and higher-binding phages panned by *M.tb*-MA. The target DNA sequences were obtained by sequencing positive phage clones. A total of 15 phage clones possessed the same sequence (GAQTCMN, marked as APTX4870; Figures 1A,B). To detect the binding intensity, *M.tb*-MA, or APTX4870 was coated onto ELISA plates at different concentrations. The wells were then filled with His-tagged APTX4870 or *M.tb*-MA, and absorbance was measured. The binding of phage clones (Figure 1C) and His-tagged APTX4870 (Figure 1E) to *M.tb*-MA increased with concentration. *M.tb*-MA with his tag could attach to coated phage clones (Figure 1D), and the binding was concentration-dependent; nevertheless, the absorbance was much lower than that of His-tagged APTX4870 binding to coated *M.tb*-MA. These results indicate that APTX4870 can strongly bind to *M.tb*-MA.

### The active sites of *M.tb*-MA-specific binding peptide have been identified.

**Figure 2:**
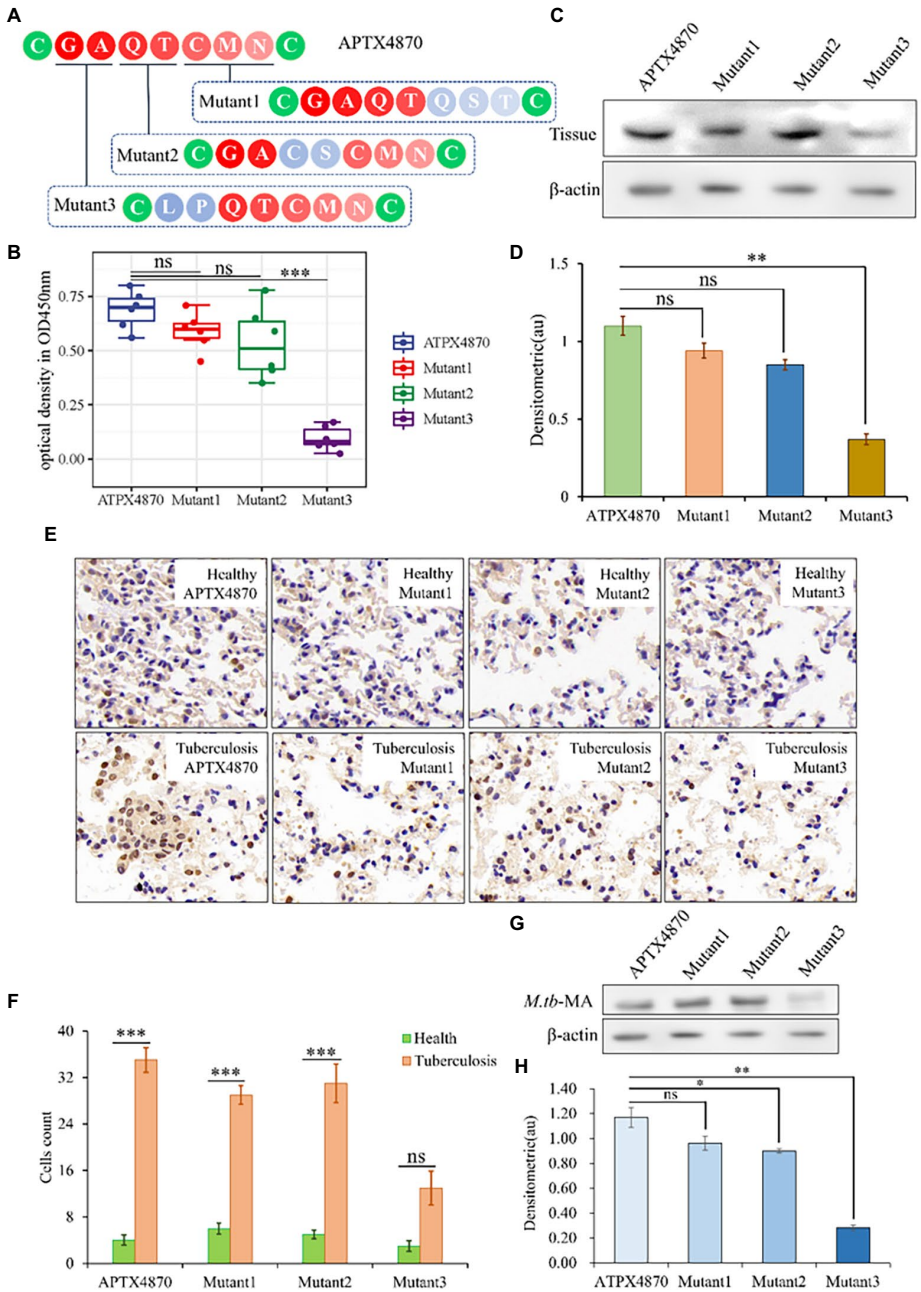
Identification of the active sites of *M.tb*-MA specific binding peptide. **(A)** Schematic diagram of APTX4870 amino acid mutations. **(B)** The binding of different His-mutants to patients lung tissue was tested *via* ELISA. The supernatant was coated on an ELISA plate for antibody sandwich detection. Custom antibodies were designed against APTX4870 (1:1000), Mutant1 (1:1000), Mutant2 (1:1000), and Mutant3 (1:1000) using Custom Polyclonal Antibody (*n* = 6). **(C)** The binding ability of different mutants to the lung tissue of tuberculosis patients was detected by WB (*n* = 3). **(D)** Semi-quantitative expression of indicated proteins by optical densitometry assay using ImageJ Software version 1.46. Data were presented as mean ± SD, **: 0.01 < *p* < 0.05: Mutant3 vs. APTX4870 (*n* = 3). **(E)** The binding ability of different mutants to the lung tissue of tuberculosis patients was detected by IHC. Scale bar = 50 μm, *n* = 6 per group. **(F)** Quantitative the expression of binding sites by optical densitometry assay using ImageJ Software version 1.46. **(G,H)** Detection of binding of mutant polypeptides to M.TB-MA in cells. *n* = 3 per group. Quantitative the expression of binding sites by optical densitometry assay using ImageJ Software version 1.46. The data were shown as means ± SD and compared by a 2-way ANOVA test. *p < 0.05; ***p < 0.01: Tuberculosis vs. Health.

We altered each *M.tb*-MA-binding site in APTX4870 separately to establish their functional importance. The binding ability of APTX4870 to *M.tb*-MA disappeared following mutation of its first and second amino acids: leucine and proline (Figures 2A–F). The specificity of APTX4870 binding to *M.tb*-MA was further examined by immunohistochemistry of clinical samples. Human lung samples were incubated with His-APTX4870 wild-type or His-APTX4870 mutants overnight at 4°C. Tissue proteins were detected by WB using an antibody against His-ATPX4870. Immunohistochemistry and WB demonstrated that His-APTX4870 could bind to lung tissues of TB patients and not of normal individuals. Mutations of leucine and proline, the first and second amino acids, in APTX4870 significantly decrease its binding. After WB detection, we found similar results in cell experiments and clinical trials. Mutations of leucine and proline, the first and second amino acids, in APTX4870 significantly decrease its binding with *M.tb*-MA (Figures 2G,H). As a result, leucine and proline residues in APTX4870 are required for binding to *M.tb*-MA. APTX4870 inhibits *M.tb*-MA-induced cell injury *in vivo*.

**Figure 3:**
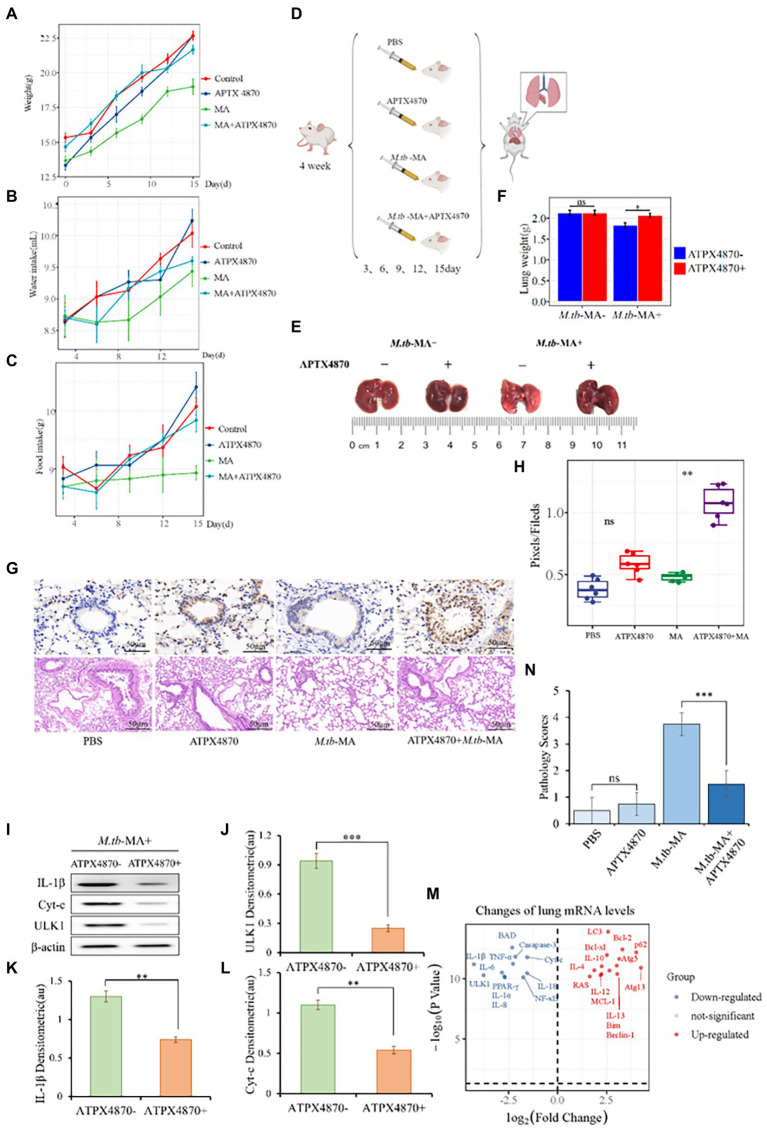
APTX4870 inhibits *M.tb*-MA induced cell injury *in vivo*. **(A–C)** The measurement of weight, food intake, and drinking water of mice in different treatment groups (*n* = 6). Body weights of mice were measured on days 0, 3, 6, 9, 12, and 15. Food consumption in mice was measured on days 3, 6, 9, 12, and 15. Following the placement of 50 g of feed on the 0th day, the food intake of the mice was estimated on the 3rd day by weighing the decreased grams of feed, and so on. The procedure for measuring mice’s water consumption is the same as for eating. **(D)** Treatment scheme for different groups. **(E,F)** Representative images of mice lung tissue and weight. The data were shown as means ± SD (*n* = 6). **(G,H)** Immunohistochemical (IHC) analysis of IL-1β, Hematoxylin, and eosin (H&E) staining of the lungs. Scale bars, 50 μm. Quantitative the expression of IL-1β by optical densitometry assay using ImageJ Software version 1.46. The data were shown as means ± SD and compared by a 2-way ANOVA test. **0.01 < *p* < 0.05: APTX4870 + *M.tb*-MA vs. *M.tb*-MA (*n* = 6; **I–L**). Representative immunoblots of IL-1β, ULK1, and Cyt-c in mice lungs from three repeated experiments. Semi-quantitative expression of indicated proteins by optical densitometry assay using ImageJ Software version 1.46. Data were presented as mean ± SD, **0.01 < *p* < 0.05; ***p < 0.001: APTX4870 + *M.tb*-MA vs. *M.tb*-MA (*n* = 3). **(M)** Total RNA was extracted from lung tissue in order to assess gene expression. The result of the APTX4870 co-treated group vs. the *M.tb*-MA group was shown by Volcano. *p < 0.05, log2 Fold Change ≥2 (*n* = 3). **(N)** The severity of per bronchial inflammation was graded quantitatively by the five-point scoring system (*n* = 6).

We administered APTX4870 and/or *M.tb*-MA to BALB/c mice. *M.tb*-MA-treated animals had reduced hunger, water intake, and weight (Figures 3A–F). Immunohistochemical analysis revealed increased IL-1β levels in *M.tb*-MA-treated groups compared with the control and APTX4870-treated groups. The co-treatment of APTX4870 and *M.tb*-MA resulted in a reduced IL-1β expression (Figures 3G,H). HE staining showed normal pulmonary tissues in mice in the control group (Figures 3G,N). The *M.tb*-MA group presented severe histopathological changes along with a mass of infiltrating inflammatory cells, pulmonary traps, and pulmonary cell extinctions (Figures 3G,N). The pulmonary histopathological tissues from the co-treated group were intact and thin, did not show immune infiltration, and the structure of the bronchioles was intact and clear.

APTX4870 co-treatment reduced the expression of IL-1, ULK1, and cytochrome c in the lungs of the *M.tb*-MA group (Figures 3I–L). The mRNA expression profiles of the co-treated group vs. *M.tb*-MA group are presented using Volcano plot (Figure 3M). APTX4870 co-treatment decreased the mRNA levels of pro-apoptotic and pro-inflammatory factors while increasing the mRNA levels of pro-autophagic, anti-apoptotic, and anti-inflammatory factors. The results of western blotting corroborated the RT-qPCR results.

APTX4870 attenuates the decrease in cell viability induced by *M.tb*-MA and protects mitochondrial integrity.

**Figure 4:**
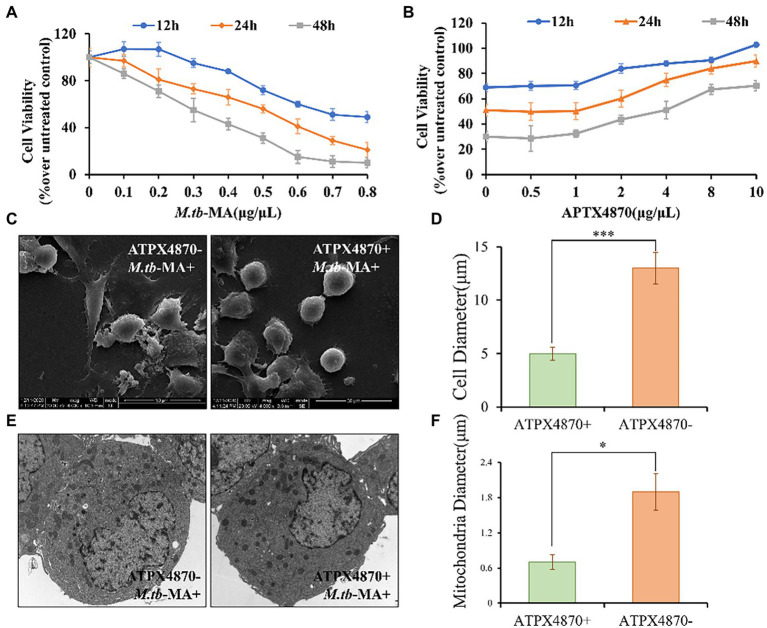
APTX4870 attenuates the decrease in viability and guards mitochondrial integrity induced by *M.tb*-MA. **(A)** The changes of THP-1 cell viability after being infected with *M.tb*-MA at a concentration of 0-1 μg/μL for 12 h, 24 h, 48 h. Data were presented as mean ± SD (*n* = 6). **(B)** The effects of APTX4870 were co-treated with 0.5 μg/μL *M.tb*-MA for 12 h, 24 h, and 48 h. Data were presented as mean ± SD (*n* = 6). **(C)** Scanning electron micrographs of THP-1 cells (SEM). **(D)** THP-1 cells diameter measurements in different groups (*n* = 6). Data were presented as mean ± SD. ***p < 0.001: *M.tb*-MA vs. APTX4870 co-treated group. **(E)** Analysis of cell morphology by transmission electron microscopy (TEM). **(F)** Mitochondrial diameter measurements in different groups (*n* = 6). Data were presented as mean ± SD. *p < 0.05: *M.tb*-MA vs. APTX4870 co-treated group.

Cell viability was detected using MTT assay. Cells were incubated for 12, 24, and 48 h with different *M.tb*-MA concentrations (0–1 μg/μL). The dose-dependent cytotoxicity of *M.tb*-MA was evident on THP-1 cells (Figure 4A). A significant reduction of cell viability was seen at 0.5 μg/μL *M.tb*-MA treatment for 12 h (*p* < 0.001), which was further reduced after 24 and 48 h compared to that of the untreated control (p < 0.001). Therefore, 0.5 μg/μL concentration was selected for subsequent studies. Co-treatment of APTX4870 at different concentrations (0–10 μg/μL) with 0.5 μg/μL *M.tb*-MA significantly increased cell viability (Figure 4B). Therefore, in the subsequent experiment, 1 μ g/μ L was chosen as the concentration of ATPX 4870.

SEM research revealed that *M.tb*-MA may disrupt the continuity of the cell membrane, resulting in membrane breakdown and cytoplasmic leakage in THP-1 cells. However, THP-1 cells were intact with normal microvilli in the APTX4870-*M.tb*-MA co-treated group (Figures 4C,D). TEM was used to assess the morphology of organelles, particularly mitochondria. TEM revealed that mitochondrial biogenesis was decreased in *M.tb*-MA-treated cells, as shown by a lower number of mitochondria and enlarged mitochondria with disordered cristae (Figures 4E,F). However, in the APTX4870-*M.tb*-MA co-treated group, the mitochondrial inner and outer membranes were intact, the mitochondrial raft structure was evident, and continuity was preserved. APTX4870 reduces autophagic inhibition induced by *M.tb*-MA.

**Figure 5:**
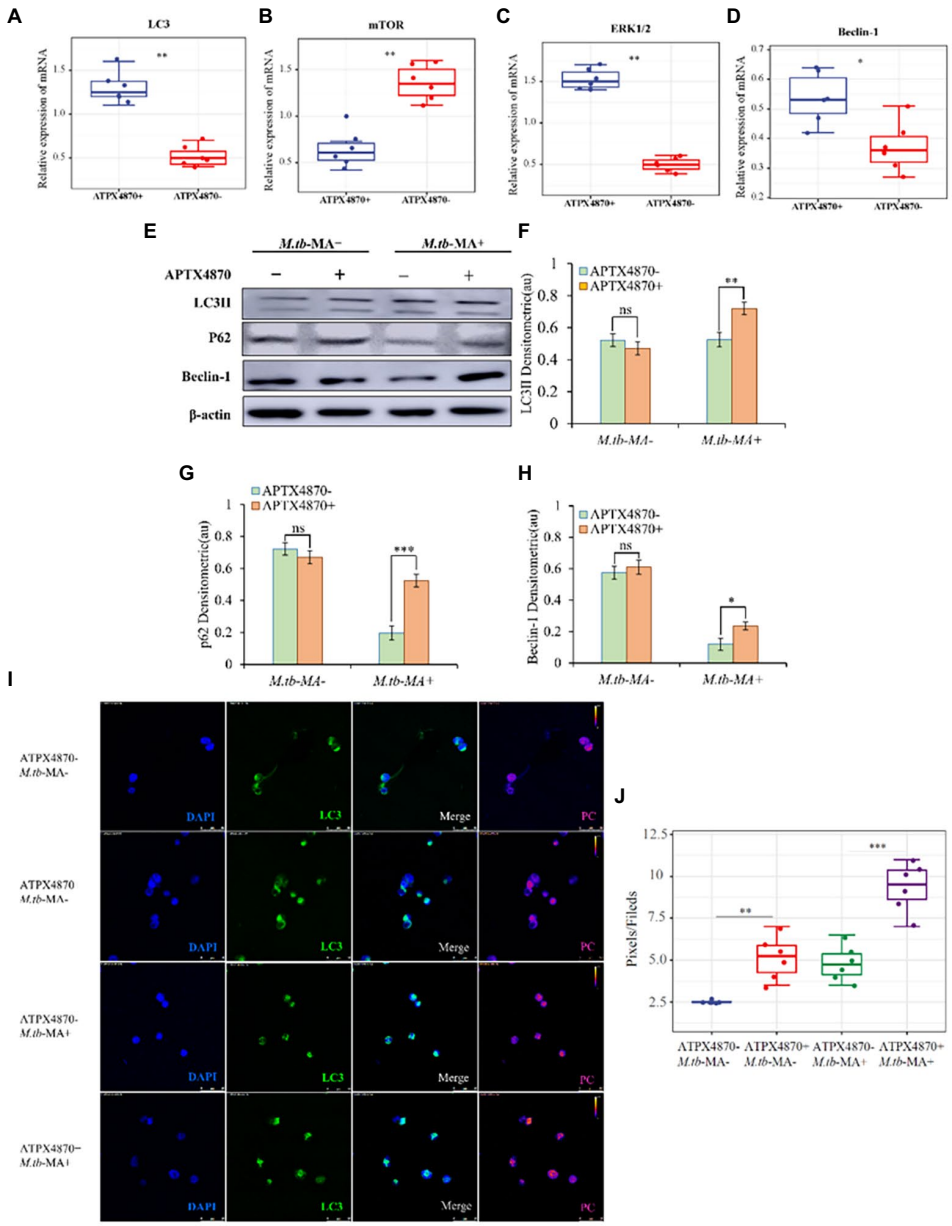
APTX4870 could reduce the inhibiting of autophagy induced by *M.tb*-MA. **(A–D)** Effects of APTX4870 co-treated induced activation of autophagy cytokines activation was measured by q-PCR. Change of secreted cytokines content of (A) LC3, **(B)** mTOR, **(C)** ERK1/2, **(D)** Beclin-1 (*n* = 6). Data were presented as mean ± SD, *p < 0.05; **0.01 < p < 0.05: APTX4870+ *M.tb*-MA vs. *M.tb*-MA. **(E–H)** Immunoblots of LC3, mTOR, and Beclin-1 in different groups. Semi-quantitative expression of indicated proteins by optical densitometry assay using ImageJ Software version 1.46 (*n* = 3). Data were presented as mean ± SD, *p < 0.05; **0.01 < *p* < 0.05; ***p < 0.001: APTX4870+ *M.tb*-MA vs. *M.tb*-MA. **(I,J)** The expression of LC3 was analyzed by immunofluorescence cytochemistry. LC3 is visualized in green and DAPI-stained nuclei in blue. Pseudo-color is used to show the difference in fluorescence intensity. Scale bars: 50 μm. Fluorescence values were quantitatively analyzed 6 images and boxplots were drawn. Quantitative the expression of LC3 by optical densitometry assay using ImageJ Software version 1.46. Data were presented as mean ± SD, *p < 0.05; **0.01 < *p* < 0.05; ***p < 0.001: APTX4870+ *M.tb*-MA vs. *M.tb*-MA.

To measure the changes in autophagy in different groups, protein and mRNA levels of autophagic indicators were detected in THP-1 cells. The co-treated group showed increased mRNA levels of LC3, Beclin-1, and ERK1/2, while the level of mTOR was reduced (Figures 5A–D). Western blotting was used to detect autophagy proteins, which confirmed the mRNA expression data (Figures 5E–H). Immunofluorescence and WB indicated a significantly higher expression of LC3 in the co-treated group than that in *M.tb*-MA-treated group (Figures 5I,J).

APTX4870 reduced ROS-dependent cell death mediated by *M.tb*-MA infection *in vitro*.

**Figure 6:**
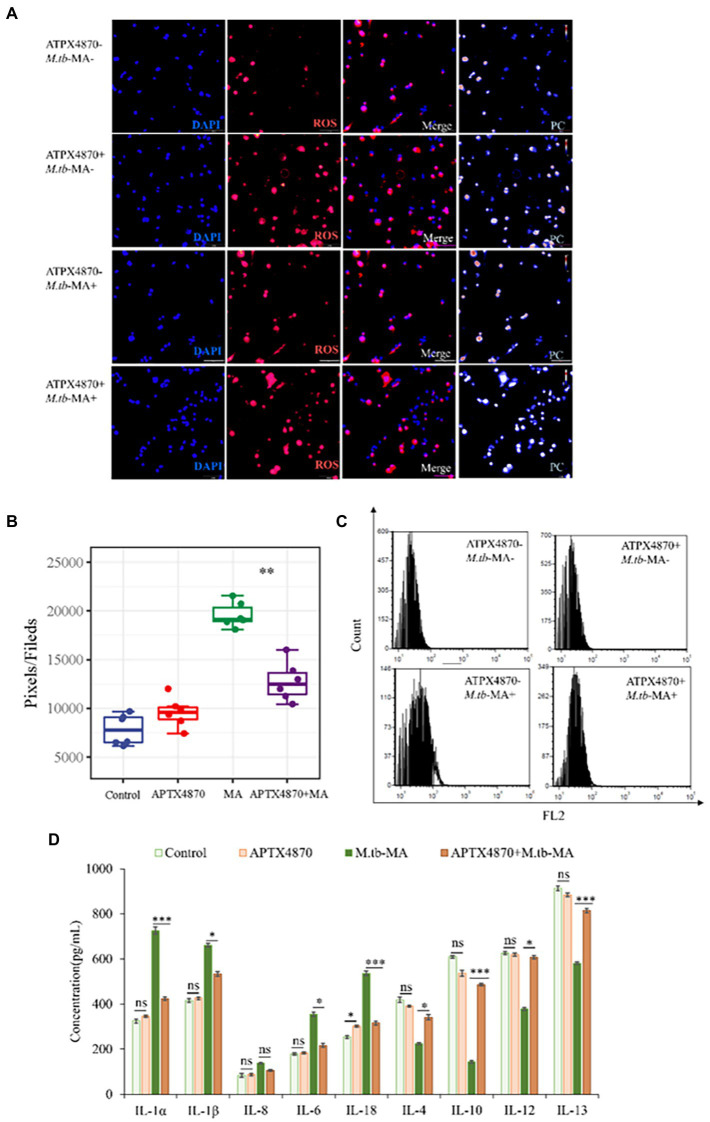
ROS-dependent cell apoptosis induced by *M.tb*-MA infection could be inhibited by APTX4870 *in vitro*. **(A,B)** The expression of ROS was analyzed by immunofluorescence cytochemistry. ROS is visualized in red and DAPI-stained nuclei in blue. Pseudo-color is used to show the difference in fluorescence intensity. Scale bars: 50 μm. Results from one experiment, representative of 6 independent experiments (*n* = 6), are shown. Quantitative the expression of ROS by optical densitometry assay using ImageJ Software version 1.46. Data were presented as mean ± SD, *p < 0.05; **0.01 < *p* < 0.05; ***p < 0.001: APTX4870+ *M.tb*-MA vs. *M.tb*-MA. **(C)** Flowcytometric analysis of total ROS accumulation (*n* = 3). **(D)** The concentration of pro-inflammatory factors and anti-inflammatory factors was measured through ELISA (*n* = 3). Data were presented as mean ± SD, compared with uninfected controls, *p < 0.05; **0.01 < *p* < 0.05; ***p < 0.001: APTX4870+ *M.tb*-MA vs. *M.tb*-MA.

Using immunofluorescence assays (Figure 6A), full-band microplate reader assays (Figure 6B), and flow cytometry (Figure 6C), we investigated the impact of APTX4870 on ROS production. Immunofluorescence and flow cytometry revealed that the *M.tb*-MA-treated group produced more ROS (Figures 6A–C). Co-treatment with APTX4870, on the other hand, lowered intracellular ROS levels. ELISA research revealed that *M.tb*-MA therapy decreased anti-inflammatory factors while increasing pro-inflammatory factors; however, the co-treated group had the reverse effect (Figure 6D). APTX4870 inhibits the binding of *M.tb*-MA to Nlrx1.

**Figure 7:**
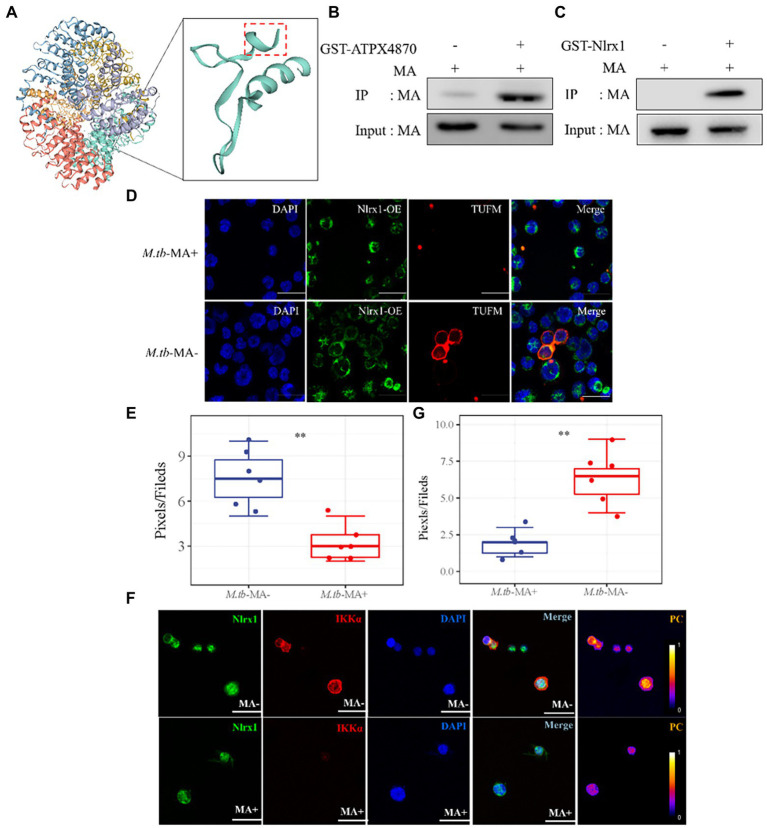
Peptides exert function by inhibiting the binding of *M.tb*-MA and Nlrx1. **(A)** Schematic of Nlrx1 protein structure and the APTX4870 homologous sequence. **(B,C)** GST-pull down assay. GST-APTX4870 or GST-Nlrx1 was incubated with *M.tb*-MA for 3–5 h and then pulled down with GSH beads. The precipitates were subjected to immunoblot and probed with *M.tb*-MA antibody (*n* = 3). **(D,E)** The expression of Nlrx1 and TUFM was analyzed by immunofluorescence cytochemistry. Nlrx1 was visualized in green, TUFM was visualized in red and DAPI-stained nuclei in blue. Scale bars: 50 μm. Results from one experiment, representative of three independent experiments (*n* = 6) are shown. Quantitative the expression of TUFM by optical densitometry assay using ImageJ Software version 1.46. Data were presented as mean ± SD, **0.01 < *p* < 0.05: *M.tb*-MA+ vs. *M.tb*-MA-. **(F,G)** The expression of Nlrx1 and IKKα was analyzed by immunofluorescence cytochemistry. Nlrx1 was visualized in green, IKKα was visualized in red and DAPI-stained nuclei in blue. Scale bars: 50 μm. Results from one experiment, representative of 3 independent experiments (*n* = 6) are shown. Quantitative the expression of IKKα by optical densitometry assay using ImageJ Software version 1.46. Data were presented as mean ± SD, **0.01 < *p* < 0.05: *M.tb*-MA+ vs. *M.tb*-MA-.

To find potential *M.tb*-MA binding partners, sequence alignment was done, which indicated that the APTX4870 sequence was comparable to that of human Nlrx1 at the 93–281 bp region (Figure 7A). Nlrx1 promotes autophagy by recruiting autophagy-related proteins and regulates the activation of the NLRP3 inflammasome to attenuate apoptosis. The binding between *M.tb*-MA and Nlrx1 was analyzed by GST pull-down (Glutathione S transferase) experiments. Results suggested that *M.tb*-MA specifically interacted with Nlrx1 (Figures 7B,C). The co-expression of Nlrx1 and TUFM was evaluated using immunofluorescence. TUFM and Nlrx1 were stained with red and green fluorescent tags, respectively. The merged photomicrographs showed the overlap of red and green fluorescence in *M.tb*-MA-untreated group (Figures 7D,E). We also detected the co-expression of Nlrx1 and IKKα by immunofluorescence. IKKα and Nlrx1 were stained with red and green fluorescent probes, respectively. The merged microphotographs showed the overlap of red and green fluorescence in *M.tb*-MA-untreated group (Figure 7F). These results indicated that *M.tb*-MA suppressed Nlrx1 expression and subsequently downregulated downstream protein expression.

## Discussion

Mycolic acid, a carbon metabolism intermediary in *M.tb*, is required for intracellular colonization and accumulation (Dookie et al., 2018; de Martino et al., 2019). It is important in the pathogenesis, toxicity, and persistence of *M.tb* and can be utilized as a target for anti-tuberculosis medications. (Cumming et al., 2018; Chai et al., 2019). *M.tb*-MA induces inflammation, causing pulmonary oxidative damage in hosts (Amaral et al., 2019; Hackett et al., 2020). We used phage display method to create a high-specificity *M.tb*-MA-binding peptide and chose APTX4870 after peptide sequence alignment, screening with three different peptide stocks, and bioinformatic analysis. The active site was subsequently discovered using precise amino acid alterations, and the cyclic heptapeptide’s primary stochastic conformation was simulated. APTX4870’s binding effectiveness to *M.tb*-MA, as well as its antiapoptotic and autophagy-inducing capabilities, were investigated. Mycolic acid induces host inflammation, while *M.tb* colonization and accumulation in cells during infection produces pulmonary tissue injury (Tomita et al., 2019; De la Cruz-Villegas et al., 2020). APTX4870 treatment alleviated pulmonary tissue injury induced by *M.tb*-MA. This may be attributed to the binding between APTX4870 and MA.

APTX4870 improved cell survival while also protecting against MA-induced cellular and mitochondrial damage. Recent studies have suggested the vital role of dynamic balance between autophagy and apoptosis in host survival (Kemp, 2017; Song et al., 2017; Chen et al., 2019). Mycolic acid has been found to suppress autophagy and promote apoptosis. In the present study, autophagy-related proteins, including LC3, Beclin-1, and ERK1/2, were suppressed by *M.tb*-MA treatment. APTX4870 and *M.tb*-MA co-treatment reversed these trends and lowered intracellular ROS generation, which reduced oxidative stress and host cell damage (Vijay et al., 2018; Kim et al., 2020).

**Figure 8:**
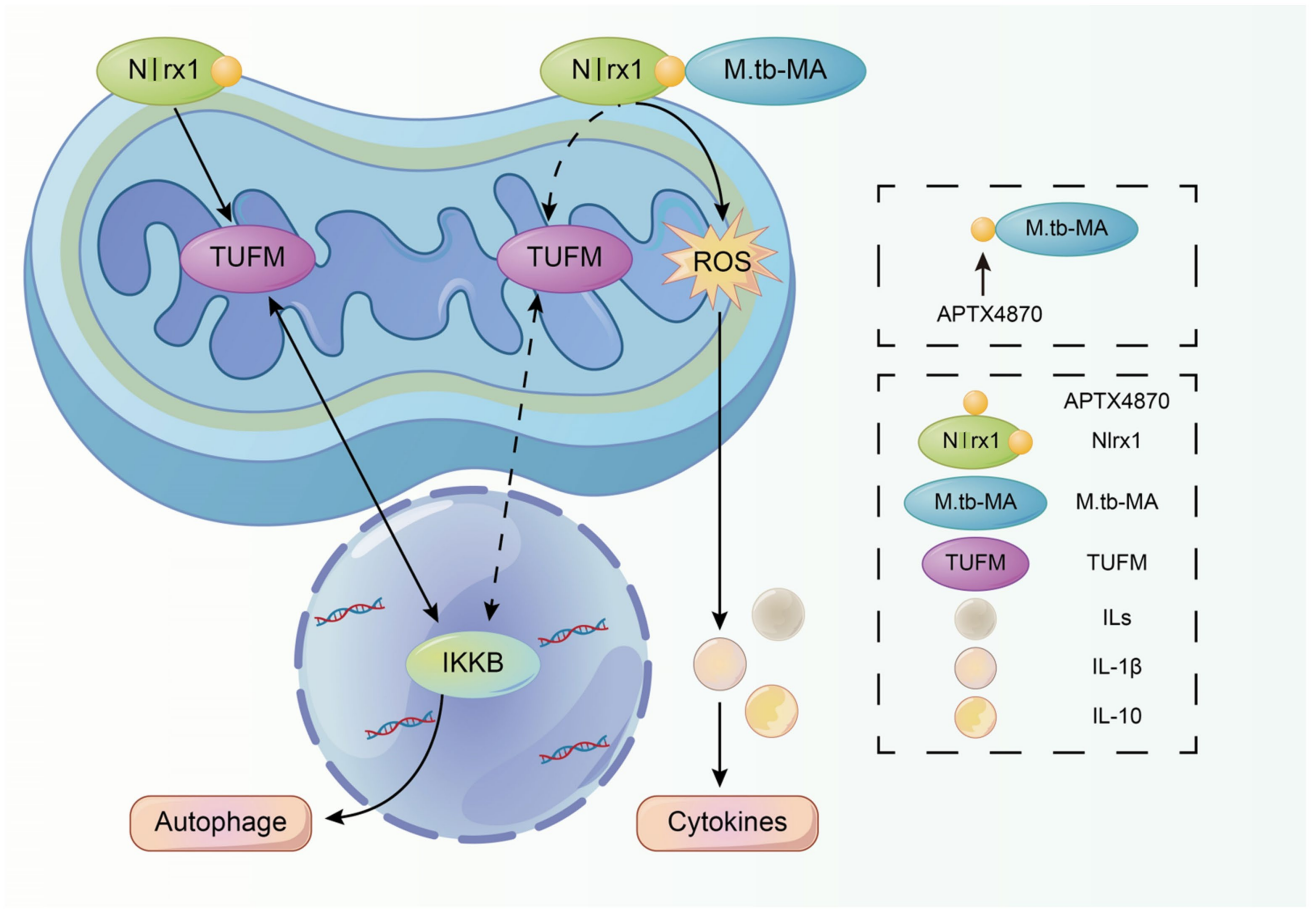
Schematic diagram of the mechanism of APTX4870.

Furthermore, we identified Nlrx1, a protein homologous to APTX4870, as a target of *M.tb*-MA. Nlrx1 has been reported to promote autophagy by interacting with TUFM and regulating the nuclear protein IKKα to facilitate the recruitment of autophagy-related proteins. We observed that *M.tb*-MA suppressed IKKα expression. As a result, *M.tb*-MA disrupts the dynamic balance between autophagy and apoptosis, most likely by targeting Nlrx1 and decreasing IKK expression, whereas APTX4870 binding to *M.tb*-MA leads to a reduction in host damage (Figure 8).

## Data availability statement

The raw data supporting the conclusions of this article will be made available by the authors, without undue reservation.

## Ethics statement

The studies involving human participants were reviewed and approved by the Ethics Committee of General Hospital of Ningxia Medical University. The patients/participants provided their written informed consent to participate in this study.

The animal study was reviewed and approved by the Ethics Committee of Ningxia University.

## Author contributions

XL, WJ, ZL, GF, WL, YS, YK, and CZ made substantial contributions to the conception and design, acquisition of data or analysis, and interpretation of data. ZL and DX drafting the article, reviewing/revising it critically for important intellectual content and final approval of the version to be published. All authors contributed to the article and approved the submitted version.

## Funding

This study was funded by the Key Project of Research and Development of Ningxia Hui Autonomous Region of China (Nos. 2020BEG03019 and 2022BEG03126), Natural Science Foundation of Ningxia (Nos. 2022AAC03029, 2021AAC03109, 2021AAC03396, 2022AAC03548, and 2022AAC03470),and Key Project of Research and Development of Ningxia Hui Autonomous Region of China (No. 2021BEG03090).

## Conflict of interest

The authors declare that the research was conducted in the absence of any commercial or financial relationships that could be construed as a potential conflict of interest.

## Publisher’s note

All claims expressed in this article are solely those of the authors and do not necessarily represent those of their affiliated organizations, or those of the publisher, the editors and the reviewers. Any product that may be evaluated in this article, or claim that may be made by its manufacturer, is not guaranteed or endorsed by the publisher.

## Footnotes

^1^ http://rsbweb.nih.gov/ij/

^2^ https://hiplot-academic.com/?lang=zh_cn
